# Visible neural networks for multi-omics integration: a critical review

**DOI:** 10.3389/frai.2025.1595291

**Published:** 2025-07-17

**Authors:** David Antony Selby, Rashika Jakhmola, Maximilian Sprang, Gerrit Großmann, Hind Raki, Niloofar Maani, Daria Pavliuk, Jan Ewald, Sebastian Vollmer

**Affiliations:** ^1^Data Science and its Applications, German Research Center for Artificial Intelligence (DFKI), Kaiserslautern, Germany; ^2^School of Computation, Information and Technology, Technical University of Munich, Munich, Germany; ^3^Department of Dermatology, University Medical Center of the Johannes Gutenberg-University Mainz, Mainz, Germany; ^4^College of Computing, University Mohammed VI Polytechnic, Ben Guerir, Morocco; ^5^Department of Computer Science, University of Kaiserslautern–Landau (RPTU), Kaiserslautern, Germany; ^6^Center for Scalable Data Analytics and Artificial Intelligence (ScaDS.AI) Dresden/Leipzig, Leipzig University, Leipzig, Germany

**Keywords:** multi-omics integration, deep learning, explainable AI, machine learning, interpretable models, gene regulatory networks, pathways, neural networks

## Abstract

**Background:**

Biomarker discovery and drug response prediction are central to personalized medicine, driving demand for predictive models that also offer biological insights. Biologically informed neural networks (BINNs), also referred to as visible neural networks (VNNs), have recently emerged as a solution to this goal. BINNs or VNNs are neural networks whose inter-layer connections are constrained based on prior knowledge from gene ontologies and pathway databases. These sparse models enhance interpretability by embedding prior knowledge into their architecture, ideally reducing the space of learnable functions to those that are biologically meaningful.

**Methods:**

This systematic review-the first of its kind-identified 86 recent papers implementing BINNs/VNNs. We analyzed these papers to highlight key trends in architectural design, data sources and evaluation methodologies.

**Results:**

Our analysis reveals a growing adoption of BINNs/VNNs. However, this growth is apparently juxtaposed with a lack of standardized, terminology, computational tools and benchmarks.

**Conclusion:**

BINNs/VNNs represent a promising approach for integrating biological knowledge into predictive models for personalized medicine. Addressing the current deficiencies in standardization and tooling is important for widespread adoption and further progress in the field.

## 1 Introduction

High-throughput technologies have transformed biological research, enabling the collection of large-scale data from different molecular layers and leading to the emergence of multi-omics, an approach that combines information from diverse sources, such as genomics, transcriptomics, proteomics, and metabolomics (Sun and Hu, 2016). This allows researchers to analyse complex interactions and regulatory mechanisms that drive cellular function, disease progression and response to therapeutic interventions, with the potential to identify novel biomarkers and better understand how genetic and environmental factors influence disease (Subramanian et al., 2020).

However, multi-omics analysis is fraught with challenges due to high dimensional, heterogeneous data, requiring methods capable of modeling non-linear relationships across multiple processes. Modern machine learning (ML) techniques, such as deep learning, offer superior predictive capabilities at the expense of interpretability. In this context, even explainable AI (XAI) metrics may lack clear or robust biological interpretations (Hancox-Li, 2020; Slack et al., 2021; Wei et al., 2024).

**Figure 1:**
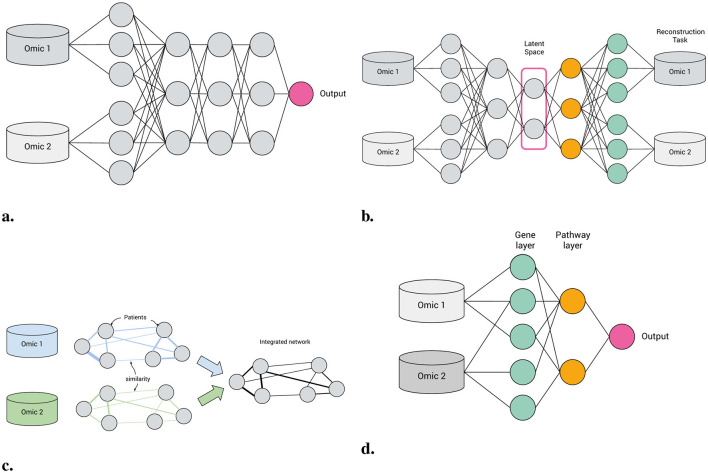
Machine learning approaches to multi-omics integration. **(a)** Feedforward neural network. **(b)** Autoencoder. **(c)** Patient similarity network. **(d)** Biologically-informed, visible neural network.

To meet this demand, visible neural networks (VNNs), also known as biologically-informed neural networks (Binns), have gained prominence (Selby et al., 2025). Unlike conventional neural networks (NNs), which learn relatively unconstrained functional approximations, VNNs incorporate prior knowledge directly into their architecture: previously “hidden” nodes map directly to entities such as genes or pathways, with inter-layer connections constrained by their ontology (see Figure 1).

VNNs have the potential to enhance biomarker discovery and drug development by learning relationships between genes, pathways, drugs and disease phenotypes. Various VNN models have been proposed, but standard terminology and clear design practices—fostering scientific reproducibility, robustness and generalizability—do not yet exist.

Several existing survey papers offer a high-level overview of interpretable deep learning in omics, but without focussing on detailed comparisons of biologically-informed architectures. This review *focusses specifically on VNNs*, filling an important gap in the literature. We examine design considerations in constructing different VNN architectures and applying them to different tasks and data sources.

Our contributions are as follows: a taxonomy of Binn architectures, a critical appraisal of the dependencies, assumptions, data sources and tools involved in building Binns/VNNs; and identification of several research gaps and future work. We consider three underlying research questions:

1. What advantages do Binns offer over traditional ML models, and how is their relative performance typically benchmarked?
2. Are biological interpretations from VNNs robust to architectural design decisions and reproducible across studies?
3. Can VNNs uncover new scientific knowledge?

## 2 Background

A living cell is a complex system of interacting molecules, where metabolites and energy are used to form biomass. The cell's regulation is guided by the central dogma of biology: DNA encodes RNA, which, in turn, encodes proteins (Crick, 1970). RNA and proteins both play roles in metabolism, structure, replication, and other cellular processes, interacting with each other and with DNA. These interactions can regulate cellular functions, for example as recruiting transcription factors or opening chromatin in eukaryotes (Kadonaga, 1998). Gene interactions have been extensively studied, expanding our understanding of cellular interaction networks, or *pathways* (Yeger-Lotem et al., 2004). Pathways connect entities such as genes, proteins and metabolites with other tissue components, reflecting cellular functions from simple growth to complex immune response. Databases describing such molecular interactions are publicly available (see Section 5) representing decades of biomedical research into causal relationships.

Multi-omics analysis uses data from heterogeneous sources to describe biological processes (Hasin et al., 2017; Subramanian et al., 2020). Modeling such sensitive and expensively collected data necessitates modern statistical methods that exploit existing domain knowledge to solve the “small data” problem (Rahnenführer et al., 2023). Biomarker discovery in single-omics data often relies on regularized linear models fit to a target of interest. Biomarkers are derived from signals of significant features, with enrichment analysis providing insights into their biological functions, or the features with largest coefficients identified as targets for validation in the laboratory (Ng et al., 2023).

Explainable AI (XAI) is a growing field of research that aims to produce methods to explain the reasoning behind ML models' predictions. *Ante-hoc* explainability refers to models that are intrinsically interpretable, whilst *post-hoc* methods are a way of gaining insights about “black box” model predictions in terms of their inputs (Retzlaff et al., 2024). Statistical models (or “data models”; Breiman, 2001), such as generalized linear models, are preferred when the main goal is inference: understanding relationships between variables that describe a natural process. On the other hand, when accurate prediction is a priority, especially when datasets are large and high-dimensional, black-box models (“algorithmic modelling”), are preferred, and capable of learning signals from data with intrinsic structure, such as images and sequences. However, larger samples and computational resources are necessary for such methods to outperform simpler models on tabular data (Grinsztajn et al., 2022).

To simplify the learning process, the search space can be constrained through provision of additional information from prior domain knowledge. Many taxonomies exist for these methods (Van Harmelen and Ten Teije, 2019; Karniadakis et al., 2021; Dash et al., 2022). Data augmentation involves generating additional data through preprocessing, e.g. varying contrast of an input image. Similarly, Yang et al. (2019) consider metabolite perturbations as input, but enrich this data through simulation. Another method involves crafting specialized loss functions for specific domains. For instance, Jia et al. (2019) uses a loss based on physical laws of energy distribution. Most relevant for this review is constraining the computational graph, creating an inductive bias in the neural network. Convolutional layers, for example, consider the dependence of neighboring pixels in an image, while neural ordinary differential equations (Chen R. T. et al., 2018) account for the structure of physical processes. VNNs also fall into this category and a taxonomy is given in Section 5.

Knowledge graphs (KGs) (Hogan et al., 2021) can integrate data from various sources, including scientific literature. Increasingly used in bioinformatics to encode and retrieve complex knowledge, KGs describe entities—including genes, proteins, pathways, diseases, and functional annotations—across multiple levels of organization (Yi et al., 2022). They are often built upon pathway databases, such as Reactome (Croft et al., 2011), Gene Ontology (The Gene Ontology Consortium et al., 2023), or Kegg (Kanehisa, 2000), that provide curated ontologies focussing on cellular processes, metabolism, interactions or signaling. These databases or networks can themselves be seen as “simple” KGs with restricted set of entities and relations. Biological KGs combined with network analysis methods offer the potential to discover or explain new relationships between drugs, genotypes and phenotypes (Chandak et al., 2023) and their embeddings have been employed in biomarker discovery (Galluzzo, 2022), multi-omics integration (Xiao et al., 2023) and drug-target discovery (Zahra et al., 2023); for example, Glue is a KG-based architecture that integrates omics features based on regulatory interactions (Cao and Gao, 2022).

Constructing a NN based on such databases is a form of knowledge-intensive ML. Integrating the most general KGs may result in very large NNs that fail to learn relevant patterns. Prior knowledge integration is a universality–generalizability tradeoff: compared to a dense neural network, a VNN's function space is reduced, losing universality but generalizing better to the real-world context of the specific biological systems of interest (Miao, 2024). In most cases, this is achieved by leveraging pathway databases such as Gene Ontology, Kegg or Reactome to inform the design of hidden layers in the network, ensuring that (some or all of) the model's internal representations align with known entities and relationships. VNNs aim to emulate signaling processes, enhancing interpretability and biological relevance, thereby accurately modeling complex systems and facilitating discovery of novel insights. As Hanczar et al. (2020) observe, this approach aims to bridge the gap between data-driven ML models and mechanistic understanding, making it particularly valuable in fields like genomics and systems biology, where omics data lack the structure of imaging or text data commonly exploited by popular deep learning frameworks.

### 2.1 What's in a name?

“Visible neural network” (VNN) is one of myriad terms to describe a NN model whose hidden layers and connections are constrained by a pre-specified ontology; terminology is far from standardized, making literature search difficult. “Visible” emphasizes interpretability without restricting to biological applications: similarly, knowledge-primed (Fortelny and Bock, 2020), knowledge-guided (Lee and Kim, 2022; Hao et al., 2022) or knowledge-based neural networks (Ciallella et al., 2021), or ontology-based autoencoders (Doncevic and Herrmann, 2023; Joas et al., 2024) allow for hierarchical structures beyond biology. “Binns” can also refer to biologically-informed neural-symbolic methods (Lagergren et al., 2020; Przedborski et al., 2021; Rodríguez et al., 2023), or biologically-inspired NNs found in connectomics (Klinger et al., 2021) or neuromorphic computing (Yamazaki et al., 2022). Here, “visibility” refers to the direct incorporation of biological pathway structures into the neural network architecture, offering intrinsic interpretability, such that VNNs sit within the broader category of Binns.

## 3 Related work

VNNs are a growing sub-field at the intersection of deep learning-based multi-omics integration, interpretable ML methods for biology, and XAI more generally. Across these areas, various survey papers have been published that mention the concept of VNNs and Binns, however, most do not discuss this area in detail. An overview of these review articles, including their scope and limitations, is given in Table 1.

**Table 1:** Existing review papers on explainable deep learning for multi-omics.

**References**	**Papers**	**Scope**	**VNNs?**	**Shortcomings**
Gazestani and Lewis (2019)	43	*Narrative*. Discusses the integration of multi-omics data with network data such as genetic interactions or co-expression data. Highlights GNNs and VNNs (namely DCell).	✓	Older paper so does not cover recent developments beyond DCell.
Crawford and Greene (2020)	62	*Narrative*. How to improve ML models in biomedicine by incorporating prior knowledge, including sequence context, gene sets and pathways, interaction or co-expression networks, ontologies and phylogenetic trees.	✓	VNNs are mentioned but specific advantages, disadvantages and opportunities are not explored in detail.
Lee and Kim (2022)	68	*Narrative*. Use of DL models to understand interactions between different molecules using prior knowledge. Divides models into weakly and strongly guided, the latter including VNNs, which incorporate Gene Ontology into their architecture.	✓	Lacks discussion on other architectural designs, data types and interpretation methods relevant to VNNs.
Leng et al. (2022)	71	*Benchmark*. Benchmark of DL models for multi-omics data fusion in cancer. Compares 6 neural networks (including 2 CNNs and 2 GNNs), 4 autoencoders and 6 VAEs on simulated and real (TCGA) cancer datasets for ML tasks.	-	Biologically-informed methods are mentioned in discussion but not included in benchmark.
Samal et al. (2022)	95	*Narrative*. Review of recent interpretable deep learning methods for drug sensitivity prediction, exploring various probing strategies.	✓	Lack of discussion on model robustness and reliability of interpretations.
Dhillon et al. (2023)	98	*Systematic*. Examines use of a broad range of traditional and deep learning-based ML methods with various omics types to identify biomarkers for cancer. Emphasizes importance of multi-omics integration and highlights increasing use of DL.	✓	Limited coverage of interpretability, no explicit focus on VNNs or methods integrating prior knowledge; prominent VNN methods not cited.
Hauptmann and Kramer (2023)	28	*Benchmark*. Benchmark of DL models for multimodal fusion in drug response prediction from multi-omics. Compares 6 existing methods and proposes a new method called Omics Stacking.	-	Prior knowledge integration not mentioned.
Novakovsky et al. (2023)	95	*Narrative*. Application of XAI techniques to DL models in genomics. Primarily focusses on post-hoc interpretation methods.	✓	Does not focus specifically on VNNs.
Wysocka et al. (2023)	42	*Systematic*. DL models for multi-omics integration in cancer research. Focus on bio-centric interpretability and integration of domain knowledge at preprocessing, architectural design and posthoc comparison stage. Identifies trends in GNNs and VNNs.	✓	Limited coverage of VNNs, without explicit definition or discussion of variations.
Abbasi et al. (2024)	74	*Systematic*. Trends in data modalities, feature engineering and AI models for survival prediction from omics data.	-	No biologically-informed methods mentioned.
Chen et al. (2024)	78	*Narrative*. Perspective on opportunities and challenges for interpretable ML methods in computational biology, discussing various IML methods and their evaluation. Briefly mentions biologically-informed neural networks like DCell and P-Net as examples of IML architectures incorporating domain knowledge.	✓	Provides a general overview of IML including examples of VNNs but does not go into detail about their specific architectures.
Sidorova and Lozano (2024)	34	*Narrative*. Compares deep learning methods for survival analysis with traditional models.	✓	Only one biologically-informed model mentioned.
Wagle et al. (2024)	28	*Narrative*. Overview of DL methods for single-cell omics analysis. Comprehensive discussion of interpretability with focus on gene regulation.	✓	Lack of explicit VNN definition or discussion of VNN-specific architectures.
van Hilten et al. (2024a)	123	*Systematic*. Considerations and challenges when designing interpretable deep learning models for genomics, including characteristics of data, model architecture and interpretation strategy.	✓	Limited depth on VNNs, no direct comparison with other methods.

We consider three categories of reviews: narrative reviews, systematic reviews and quantitative benchmarks.

### 3.1 Narrative reviews

Gazestani and Lewis (2019) presented an early review of methods of ontology integration, including methods with hierarchical layers defined by gene ontology (i.e., VNNs), as well as network propagation models, a special case of GNNs. Crawford and Greene (2020) reviewed approaches for embedding biological structures into machine learning models, including sequence encodings, network embeddings and models constrained by ontological structures. Our review specifically narrows down to VNNs that use pathways and ontologies for structured input integration. Other relevant works include Novakovsky et al. (2023) on explainable AI in genetics, and Lee and Kim (2022), which distinguishes between “weak” (e.g., data-driven GNNs) and “strong” biological guidance (e.g., structured VNNs). Wagle et al. (2024) provided a review of interpretable deep learning models specifically for single-cell omics, highlighting the importance of model transparency in this context. Chen et al. (2024) provided a broad perspective on interpretable ML in computational biology. They mention biologically informed methods and categorize them, together with attention based algorithms, as “by-design” methods, which they see as naturally interpretable. Samal et al. (2022) reviewed interpretable deep learning models for drug sensitivity prediction, exploring various “probing” strategies for examining how input data is processed within the network, including some biologically-informed approaches. A brief and accessible high-level overview of Binns is also given in Selby et al. (2025).

### 3.2 Systematic reviews

Wysocka et al. (2023) conducted a systematic review of Binns. They derived a taxonomy of pre-processing, in-processing and *post-hoc* biological interpretability. Their review spans 42 publications up to 2022, sourced from PubMed and the *Web of Science*. Focussing on oncology, it covers a range of interpretability methods, including GNNs and *post-hoc* explainability measures for conventional deep learning models. About 10 models whose architecture is “explicitly defined” by domain knowledge are included. However, the relative merits of these architectures or their underlying assumptions are not critically evaluated. Dhillon et al. (2023) offered a more general systematic review of ML methods for multi-omics-based biomarker identification—again focussing on cancer—without a specific focus on biologically-informed models like VNNs. Abbasi et al. (2024) reviewed a large number of methods for survival analysis in multi-omics, using deep learning, traditional ML and statistical methods, but not any methods for knowledge integration. A recent systematic review paper by van Hilten et al. (2024a) includes 24 VNN papers, representing probably the most complete survey to date. They compare the popularity of VNNs with other methods, noting the sparsity of VNNs allows handling a greater number of input features than computationally intensive transformer models, though conventional NNs remain the most popular approach. However, the review does not distinguish different VNN architectures or discuss specific design decisions.

### 3.3 Benchmarks

Leng et al. (2022) performed a benchmark of deep learning models for multi-omics data fusion in cancer, comparing 6 NNs (including two CNNs and two GNNs), four autoencoders and six variational autoencoders on simulated and real cancer datasets for supervised and unsupervised learning tasks. However, their analysis did not cover any NNs with biologically-informed architectures. Similarly, Hauptmann and Kramer (2023) evaluated various deep learning-based multi-modal fusion approaches in drug response prediction and proposed a new method called Omics Stacking, but did not explore any VNNs. Other works extending specific methods include benchmarks of one or more VNN approaches (e.g., Esser-Skala and Fortelny, 2023): we discuss these in Section 5.

### 3.4 The gap we fill

In summary, most existing reviews either focus broadly on multi-omics ML approaches or are limited to specific contexts such as cancer or general interpretability methods. Our systematic review addresses this gap by focussing specifically on *visible neural network* architectures. We aim to provide a more detailed analysis of technical implementations, discussing the topic of robustness and reproducibility, covering the latest works in this area. Up-to-date coverage includes recent papers from 2024, focussing on applications beyond oncology and with an examination of ways in which VNNs have been both designed, implemented and evaluated, summarizing comparisons with traditional models and with each other.

## 4 Methods

Our aim was to compare architectural design considerations in Binn-like models and identify key contributions in the field. Thus we sought papers—for which the full text was available, to understand the model structure—using NN models informed by biological ontologies, applied to tabular (multi-)omics datasets.

**Figure 2:**
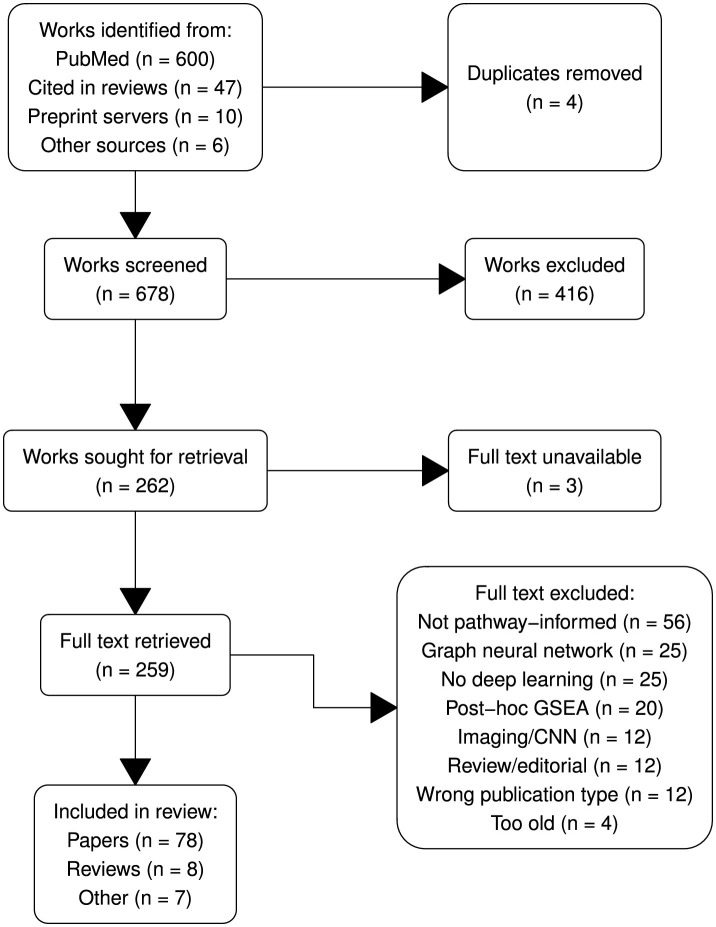
Prisma diagram summarizing article selection.

The systematic review was conducted following the Preferred Reporting Items for Systematic Reviews and Meta-Analyses (Prisma; Page et al., 2021) guidelines to ensure transparency and rigor. The Prisma flow diagram (Figure 2) outlines the process of study identification, screening, eligibility assessment and inclusion.

### 4.1 Search strategy

We conducted a comprehensive search using PubMed to identify relevant papers. Unlike Wysocka et al. (2023) we chose not to restrict our search to works in oncology, instead focussing on biologically informed models in any application with the query multi-omics AND (deep learning OR computer science OR neural networks OR network analysis OR machine learning) AND (biologically informed), filtered to works published in the period 2018–2024: spanning from the earliest known Binn papers to the most recent publications at the time of retrieval.

However, non-standard terminology in this subfield means this query alone may not capture all relevant papers. For example, Ma et al. (2018) was one of the first papers to use the term “visible neural network,” but “multi-omics” or “omic” does not appear anywhere in the article, nor do phrases like “biologically informed” or “pathway.” Other relevant works may be published in conferences or periodicals not indexed by PubMed. Rather than contorting our search query to detect these (admittedly significant) edge cases, we opted to augment our main query with a Google Scholar search for “visible neural networks” as well as adding selected papers manually, including 47 works already included in existing reviews.

Periodically, our database of results has been updated by adding new articles reported as citing key review papers, according to Google Scholar. In this way we were able to include several very recently published works, e.g., Meirer et al. (2024); Liu P. et al. (2024).

### 4.2 Screening

After removing duplicates, a total of 678 unique records were screened by title and abstract using inclusion and exclusion criteria as defined above. The screening was performed by three reviewers—with backgrounds in statistics, computer science and bioinformatics—working independently. Disagreements were resolved by majority vote, following a discussion drawing on each reviewer's respective expertise.

### 4.3 Eligibility

Full-text versions of 262 articles were assessed for eligibility against the predefined criteria, which included methodological journal or conference papers proposing neural network approaches to model omics data that integrate prior biological knowledge into their network architecture. Studies were excluded at this stage if they did not involve NNs, if they were fully connected, non-biologically informed structures, or if biological interpretations only took the form of *post-hoc* gene-set enrichment analysis (GSEA). We focussed our attention on sparsely-connected feedforward neural networks (FFNNs) and autoencoders, while convolutional, attention or graph-based models were generally excluded, as were those designed solely for genomic/proteomic sequences or imaging data.

### 4.4 Inclusion

86 papers met the eligibility criteria and were included in the final analysis. These studies represent a variety of papers proposing or evaluating biologically-informed neural network architectures, as well as several relevant survey papers (see Section 3). We also highlight 7 “honorable mentions:” papers that do not strictly meet the inclusion criteria but are interesting examples of alternative approaches.

### 4.5 Reporting

The full list of included papers is given in Appendix Table A1. The Prisma checklist is provided in the Supplementary materials.

### 4.6 Bibliometrics

**Figure 3:**
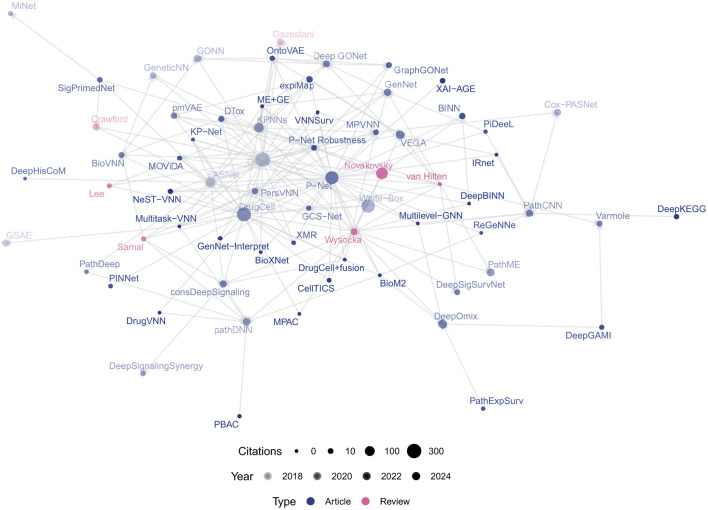
Bibliometric network of VNN papers, scaled by number of citations and shaded by year of publication.

Citation counts and relationships were retrieved from the CrossRef API using the R package **rcrossref** (Chamberlain et al., 2022) to construct a citation network between the papers (see Figure 3). We verified the existence of individual works in PubMed using the NCBI eUtils API

## 5 Results

### 5.1 Overview

Our review examines the key advancements in visual neural networks (VNNs), focussing on taxonomy, architectural innovations, methods for evaluation and practical applications. The review identified major developments across diverse architectures—feed-forward neural networks, autoencoders and graph neural networks—evaluated on a variety of omics datasets and knowledgebases. A critical theme is the tradeoff between sparsity for interpretability and the robustness of explanations in complex biological datasets. We also explore how specific design decisions, such as data integration strategies and pathway representations, influence model performance.

We consider “strong” biological guidance (Lee and Kim, 2022) where models are constrained by prior knowledge, specifically the “in-processing” paradigm (Wysocka et al., 2023) where such information informs the model's internal architecture. By contrast, wrangling data into a graph structure and applying non-dedicated ML algorithms (such as GNNs) constitutes biologically-informed “data pre-processing” (Wysocka et al., 2023) and is not our focus here; similarly we do not dwell on *post-hoc* model explanations or enrichment analyses for non-biologically-informed models (e.g. Hanczar et al., 2020).

Post-2020, VNN research has grown significantly, with key works like DCell (Ma et al., 2018), DrugCell (Kuenzi et al., 2020), and P-Net (Elmarakeby et al., 2021) central to the citation network (Figure 3). P-Net has been reproduced by Pedersen et al. (2023) and built upon by Hao et al. (2018a); Hu et al. (2022); Hartman et al. (2023), applying the framework to other modalities and targets. Similarly, MOViDA (Ferraro et al., 2023) builds upon DrugCell, itself an extension of DCell. Approximately half of recent studies are uncited by prior reviews (see Figure 2), indicating untapped contributions. A full list of retrieved works and associated abbreviations is given in Appendix Table A1.

### 5.2 Taxonomy of architectures

Table 2 divides works according to their broad architecture: feed-forward neural networks (Figure 1d) and autoencoders (Figure 1b) constitute the majority of VNNs, but there also exist some GNNs and CNNs with apparent pathway-aware properties, and other notable approaches.

**Table 2:** Architectures used in included papers (numbers of papers in parentheses).

**Architecture**	**Single omics**	**Multi-omics**
FFNN	BINN, CellTICS, Cox-PASNet, DCell, Deep GONet, DeepBINN, DrugCell, DrugCell+fusion, DTox, GeneticNN, GenNet, GenNet-Interpret, GONN, KPNNs, MPVNN, PAGE-Net, ParsVNN, PASNet, PathDeep, pathDNN, PathExpSurv, PiDeeL, PINNet, SigPrimedNet, White-Box, XAI-AGE, XMR (27)	BiGMLVQ, BioM2, BioVNN, BioXNet, Cancer-Net, consDeepSignaling, DeepGAMI, DeepHisCoM, DeepKEGG, DeepOmix, DeepSignalingSynergy, DeepSigSurvNet, DrugVNN, GCS-Net, KP-Net, ME+GE, MiNet, MOViDA, MULGONET, Multitask-VNN, NeST-VNN, P-Net, P-Net Robustness, Varmole, VNNSurv (25)
Autoencoder	ACSNI, expiMap, GONN, GSAE, OntoVAE, PAAE, pmVAE, priorVAE, VEGA (9)	PathME (1)
GNN	GraphGONet, IRnet (2)	DrugVNN, GSNNs, Multilevel-GNN (3)
CNN	PBAC, ReGeNNe (2)	PathCNN (1)
Factor Graph	FGNN (1)	MPAC (1)

A VNN is a neural network model with at least one layer of structural sparsity based on connections not directly observable in the main data. Hence, patient similarity networks (Pai and Bader, 2018, Figure 1c) are not VNNs, because their inter-node connections are derived from the data.

**Figure 4:**
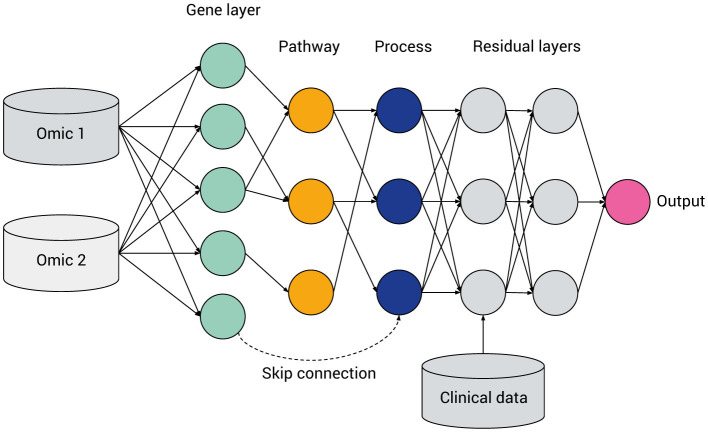
General hierarchical structure of a visible neural network, featuring multi-omics integration, a hierarchy of genes, multi-level pathways or biological processes (green, yellow, and blue, respectively), a ragged structure with skip connections and layers containing fully connected “residual” nodes. Data not mappable to pathways can be included via intermediate or late fusion.

Figure 4 gives a prototypical illustration, with various features of VNN models, described in detail later in this section and tabulated in Table 3. From left to right [input(s) to output] the model's abstraction becomes progressively more complex as later levels in the neural network correspond to higher tiers in the pathway hierarchy. Such a model can be constructed from a FFNN, with numbers of hidden layers and nodes within each layer corresponding to the desired gene and pathway levels. Initially densely connected, a masking matrix removes inter-layer connections that do not correspond to known biological relations.

- **Feed-forward networks** A “standard” NN for supervised learning, FFNNs may be applied to classification, regression or survival analysis tasks. Feed-forward VNNs may have deep structures with multiple layers of nested pathways, such as DCell (Ma et al., 2018) and P-Net (Elmarakeby et al., 2021); however the simplest networks, e.g., Cox-PASNet (Hao et al., 2018b) include just one pathway layer.
- **Autoencoders** Autoencoders are a class of deep learning models for unsupervised learning and consist of three components: an encoder, a latent space and a decoder (see Figure 1b). Input features are encoded through one or more hidden layers into a “bottleneck” layer representing a low-dimensional latent space; a decoder, typically mirroring the architecture of the encoder, then reconstructs the input features from this space. Some of the earliest Binns are autoencoders, e.g., GSAE (Chen H.-I. H. et al., 2018). A typically used extension of this idea are variational autoencoders (VAE) which are probabilistic and generative models learning a multivariate distribution (e.g., Gaussian) as latent space, enabling latent space disentanglement and compactness. Examples include Vega (Seninge et al., 2021) and ExpiMap (Lotfollahi et al., 2023); other models come in both AE and VAE variants, e.g., PAAE/PAVAE (Avelar et al., 2023), or FFNN and AE variants, e.g. GONN/GOAE (Peng et al., 2019). Liu B. et al. (2024) proposed “pathway-informed priors” where knowledge is integrated into the VAE loss term.
- **Convolutional neural networks** CNNs have been widely adopted in the analysis of structured datasets like images and sequences. Applying CNNs to multi-omics data is possible by combining with other data modalities such as sequences (DeepGo; Kulmanov et al., 2018) or by synthesizing “pathway images” from tabular data (PathCNN; Oh et al., 2021).
- **Graph neural networks** GNNs can directly leverage the topological structure of biological data, making them particularly well suited for tasks where the relationships between entities need to be explored. For example, GraphGONet (Bourgeais et al., 2022) uses the structure of the Gene Ontology graph directly; Yan et al. (2024) used a GNN to embed genes, followed by a “pathway aggregation block” resembling a FFNN, to obtain pathway-level features. As GNNs operate primarily through learned graph representations, they do not inherently enforce predefined biological hierarchies, which can make them less suitable for applications where pathway-level interpretability and strict adherence to known biological relationships are necessary.

**Table 3:** Architectural features in included papers.

**Feature**	**Value**	***n***	**Examples**
Pathway Depth	Deep	29	BINN, BioXNet, Cancer-Net, CellTICS, Deep GONet, DrugCell, DrugCell+fusion, DTox, FGNN, GCS-Net, GeneticNN, GenNet, GenNet-Interpret, GSNNs, IRnet, k-DNN, KP-Net, KPNNs, ME+GE, MOViDA, Multitask-VNN, NeST-VNN, OntoVAE, P-Net, P-Net Robustness, ParsVNN, VNNSurv, XAI-AGE, XMR
	Shallow	40	ACSNI, BiGMLVQ, BioM2, BioVNN, consDeepSignaling, Cox-PASNet, DCell, DeepBINN, DeepGAMI, DeepHisCoM, DeepKEGG, DeepOmix, DeepSignalingSynergy, DeepSigSurvNet, DrugVNN, expiMap, GONN, GraphGONet, GSAE, MiNet, MPVNN, Multilevel-GNN, PAAE, PAGE-Net, PASNet, PathCNN, PathDeep, pathDNN, PathExpSurv, PathME, PBAC, PiDeeL, PINNet, pmVAE, priorVAE, ReGeNNe, SigPrimedNet, Varmole, VEGA, White-Box
	NA	1	MPAC
Hidden Layers	✓	35	BiGMLVQ, consDeepSignaling, Cox-PASNet, DeepBINN, DeepGAMI, DeepHisCoM, DeepOmix, DeepSignalingSynergy, DeepSigSurvNet, DrugCell+fusion, expiMap, GCS-Net, GONN, GSAE, GSNNs, IRnet, MiNet, MOViDA, Multilevel-GNN, OntoVAE, PAAE, PAGE-Net, PASNet, PathCNN, PathDeep, pathDNN, PBAC, PiDeeL, PINNet, pmVAE, priorVAE, SigPrimedNet, Varmole, VEGA, VNNSurv
Mixed Layers	✓	4	BioM2, expiMap, PAGE-Net, PINNet

“Deep” means the network contains multiple biologically-informed pathway layers; “hidden” layers are made up of fully-connected nodes; “mixed” layers contain both biologically-sparse and fully-connected nodes; NA, not applicable.

Some authors adopt hybrid approaches, for example: ReGeNNe (Sharma and Xu, 2023) combines GNN and CNN layers; GraphGONet (Bourgeais et al., 2022) claims to offer advantages of an FFNN and a GNN; DeepKegg (Lan et al., 2024) introduces a “pathway self-attention module.” Biologically informed generalized matrix learning vector quantization (BiGMLVQ; Voigt et al., 2024) bills itself as a non-deep-learning approach but is nevertheless NN based. BioM2 (Zhang et al., 2024) performs pathway-level feature selection, concatenating features with filtered inputs unmapped to pathways. While this approach can be performed in a deep learning pipeline, the authors note it could also be performed using multi-stage logistic regression or other machine learning methods. In a more network focused approach, Kim et al. (2018, 2019) employ a random walk algorithm across an integrated pathway network based on the Kyoto Encyclopædia of Genes and Genomes (Kegg) pathways to derive pathway-level scores for downstream tasks such as survival prediction. However, this approach does not directly incorporate a biologically informed neural network structure. Uzunangelov et al. (2021) present Aklimate, an example of a stacked kernel learner incorporating pathway knowledge.

### 5.3 Data and applications

**Figure 5:**
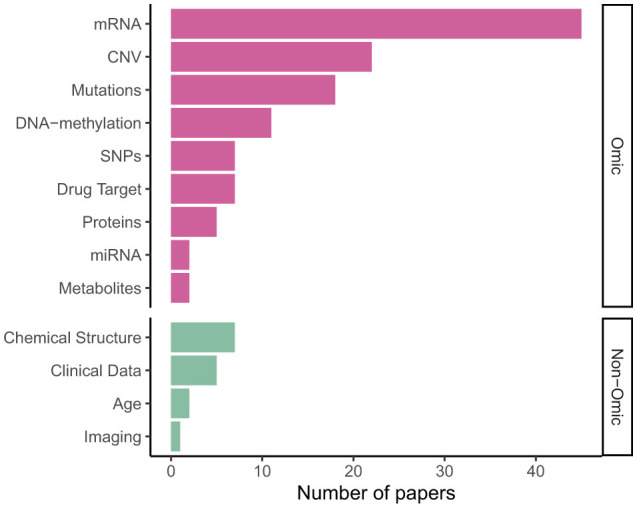
Input data types in published VNN models.

Figure 5 shows gene expression (transcriptomics/mRNA) is the dominant omics modality, featuring in more than half of papers, followed by copy number variations (CNV); DNA mutations, including single nucleotide polymorphisms (SNPs), were also a common data type. As shown in Table 2, 31 models integrated multi-omics data, with some models such as P-Net (Elmarakeby et al., 2021) ostensibly applicable to any number and combination of omics levels (in the paper itself they used just two).

A limited number of models—especially for survival analysis—integrated omics or multi-omics data with “non-omics” inputs not mappable to pathways: Cox-PASNet (Hao et al., 2018b) and MiNet (Hao et al., 2019), combine gene expression, DNA methylation and copy number variations with clinical data, such as patient age, via late fusion. DrugCell (Kuenzi et al., 2020; Greene and Costello, 2020) extended DCell (Ma et al., 2018) by integrating mutation data with embeddings of the chemical structure of different drugs, also via late fusion; however later analysis by Nguyen et al. (2024) found mid- or early fusion models performed better. Other examples of such drug–genotype modular networks include BioXNet (Yang et al., 2024), MOViDA (Ferraro et al., 2023) and XMR (Wang et al., 2023).

As shown in Appendix Table A2, oncology is a major application area for VNNs, with 46 papers; 13 were concerned with drug response (e.g., consDeepSignaling; Zhang et al., 2021); 4 with cellular processes; 3 with schizophrenia and 3 with COVID-19. While this shows cancer research is significant, VNNs are not limited to this disease.

**Figure 6:**
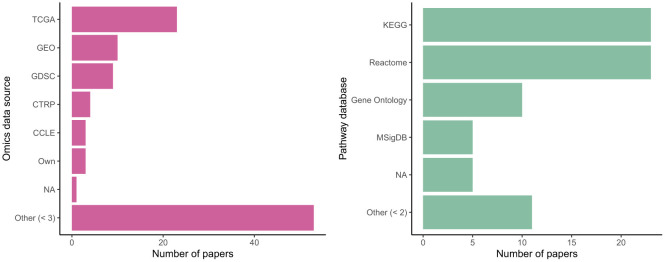
Sources of omics data and pathway knowledge in biologically-informed models.

Many authors used data from common sources, with omics data from the Cancer Genome Atlas (TCGA) and Gene Expression Omnibus (GEO) particularly popular; see Figure 6. Some authors used their own collected data or else did not clearly specify an existing database as their source. Use of common data sources may make replication of results more straightforward (nearly every paper provided analysis code: Appendix Table A4) but may also raise questions about their real-world generalizability, given sampling biases present in these cohorts. (Kegg) and Reactome were equally commonly chosen as sources of biological ontology information, followed by Gene Ontology and MSigDB. All are publicly accessible databases. Kim and Lee (2023) highlighted differences in performance for the downstream task depending on the ontology used.

### 5.4 Evaluating biologically informed architectures

Assessing the usability of VNNs requires comprehensive evaluation of predictive performance, interpretations, and robustness against appropriate baselines. Of included papers, 56 compared with traditional (non-NN) models and 54 compared with non-biologically-informed NNs, with just 22 comparing their proposed models with other Binn variants, meaning most VNNs are evaluated in isolation to other biologically-informed models.

#### 5.4.1 VNNs perform similarly to denser models

Fortelny and Bock (2020) posited that Binns operate as “information processing units” using network-based computations to regulate their states. Thus, by mirroring cellular regulatory systems, VNNs provide a deeper understanding of these mechanisms, and can offer similar prediction performance to “black box” fully-connected networks while being significantly more sparse. However, redundancy in the structure of VNNs leads to variability in edge weights, making explanations less robust—a problem that may be mitigated via dropout on hidden nodes during training. Similarly, Huang et al. (2021) argued that redundancy among nodes and edges can lead to overfitting and less robust explanations; they proposed a pruning mechanism to mitigate the issue, resulting in model simpler than DrugCell while offering superior accuracy: their ParsVNN model reduced computation time and memory footprint and number of included genes by up to 90%, with statistically significant increases in predictive performance across five datasets.

Kuenzi et al. (2020) compared their VNN to a “black-box” neural network with equivalent depth and sparsity, finding broadly similar performance and superior accuracy to a non-deep-learning model. In contrast, Pedersen et al. (2023) compared P-Net with a randomly-connected network. They observed a clear decrease in performance for the random connections in comparison to the Binn. Lin et al. (2017) also found biologically-sparse networks outperformed fully-connected models. However a fully-connected network with random masked nodes is not necessarily optimal for multi-omics if it assumes early fusion (Hauptmann and Kramer, 2023); VNNs have been shown sensitive to fusion stage (Nguyen et al., 2024), so but early-integrated baselines could be “strawmen.”

#### 5.4.2 Performance is sensitive to architecture

Several studies have compared different biologically informed architectures on the same data to assess their relative benefits. For instance, PINNet (Kim and Lee, 2023) was compared against traditional ML models and dense NNs. They also compared two types of VNNs, informed by GO and Kegg respectively, which outperformed the non-biologically-informed models. Meanwhile, PBAC (Deng et al., 2024) conducted an ablation study on its architecture, revealing that removing the biological information mask and the attention layer, respectively, reduced the performance of drug response prediction. However, they did not benchmark their architecture against other models.

#### 5.4.3 Robustness of interpretations

Since VNNs rely on the same biological knowledge as commonly used gene- or pathway-enrichment analysis frameworks, they inherit similar problems, such as balancing between specific pathways with few genes and broad pathways with many genes. Additionally, VNNs can be prone to overfitting and instability resulting from small sample sizes, leading to significant changes with different train-test splits or initializations (Esser-Skala and Fortelny, 2023). Common strategies for the training of VNNs are the introduction of dropout layers to stabilize training, in particular when overlapping ontology terms are used. Further, weights can be restricted e.g. by weight decay configuration or by restricting the direction (positive weight) to fix direction. Additionally, Esser-Skala and Fortelny (2023) assessed the robustness of interpretations in several models, including DTox and P-Net (Hao et al., 2022; Elmarakeby et al., 2021).

Meirer et al. (2024) raised the question of robustness of biomarker signatures found by Binns. Their solution, DeepBinn, fits a sub-network per pathway—each a NN with a fixed number of hidden layers—whose output weight measures that pathway's importance. By comparing the ranks of pathways over successive initializations, the authors claim to yield a “robust” signature.

### 5.5 Architectural design

Even within the taxonomies listed in Table 2, VNN architectures differ considerably, based on different design considerations highlighted in Figure 4.

#### 5.5.1 Pathway nodes

Some VNN architectures have multiple hidden nodes per gene or pathway, representing their ability to perform multiple tasks concurrently. For example, DCell (Ma et al., 2018) and derivatives (Kuenzi et al., 2020; Ferraro et al., 2023, DrugCell, MOViDA;) have 6 nodes per pathway; OntoVAE (Doncevic and Herrmann, 2023) has 3. However, most other architectures map each entity to at most one node in the network, simplifying the representation—and its interpretation—at the possible expense of predictive power.

Perhaps the most crucial design choice is the selection of the source and type of biological knowledge to be incorporated in a VNN. However, surprisingly little efforts have been made in the field to analyse and study the impact on choosing Reactome pathways, KEGG, GO-terms, or other sources. Since these sources all have their strength and weaknesses in relation to coverage (number of genes represented), overlap between terms or curation quality, a big impact on VNNs and their architectural choices is expected. For example for VAEs authors state that overlap of genes across pathways is countered by dropout layers (Seninge et al., 2021), similarly reported in feed/forward VNNs by Fortelny and Bock (2020). ParsVNN (Huang et al., 2021) approaches the problem of redundant subsystems in VNN models by a pruning mechanism.

Lastly, the chosen database or ontology, as well as the level of hierarchy, ideally matches the purpose of the model e.g., using KEGG metabolic networks for insights into cancer cell metabolism or Reactome immune pathways for studying infections. Currently, most studies using VNNs are explorative and typically do not restrict incorporated biological knowledge in a purpose-driven way. However, PathExpSurv (Hou et al., 2023) uses a “pathway expansion” phase to adaptively adjust pathway-gene connections, potentially enhancing interpretability. Also expanding on the idea of including pathway information, MPVNN (Ghosh Roy et al., 2022) is uses mutation assays to build multiple (mutated) versions of a given pathway.

#### 5.5.2 Auxiliary layers (layer-wise loss)

DCell also introduced “auxiliary layers,” a method for intermediate pathway layers (for models who have them) to contribute to the loss function and to allow attribution to particular hierarchical levels. P-Net adopts a similar approach of adding “predictive layers” with sigmoid activation after each hidden layer, with later layers weighted more highly in the loss function.

#### 5.5.3 Hidden and mixed layers

Some architectures include fully-connected hidden layers that are not *a priori* biologically interpretable. These layers introduce additional complexity to the network, potentially improving predictive power but at the expense of interpretability. For example, models like PASNet (Hao et al., 2018a) and MiNet (Hao et al., 2019) include this structure. However, the rationale behind including such layers is not always clearly explained.

The inclusion of these hidden layers may allow the model to capture latent interactions not accounted for by existing biological knowledge, but this assumption requires further validation.

Other architectures include fully-connected nodes in the same layer as the interpretable nodes, a design we will call “mixed layers;” with the the additional nodes denoted “residual” nodes for their ostensible aid to model performance or simply “hidden” nodes due to their lack of biological interpretability.

In most works the authors do not mention why they chose a given design. However, some works have performed ablation studies with and without hidden layers or nodes in the architecture: Lin et al. (2017) compare multiple dense NNs and VNNs with one or two fully connected hidden layers, seeing the best overall performance in the networks with two additional hidden layers in a clustering task. They also found pre-training not beneficial in comparison to training from scratch for clustering cells, but it increased performance for cell type retrieval (classification task). In contrast in Voigt et al. (2024), BiGMLVQ the model with more prototype layers (classification layers) performed worse, which the authors attributed to overfitting. Fortelny and Bock (2020) showed that drop-out on pathway level, as well as input layers, increased robustness to redundancies and imbalances inherent to biological networks.

In particular, autoencoders using biological knowledge to guide representation learning rely heavily on the combination of fully-connected encoders and sparse decoders representing pathway or ontology relationships. However, only authors of VEGA (Seninge et al., 2021) studied other possible architectures and combinations while others like expiMap (Lotfollahi et al., 2023) or OntoVAE (Doncevic and Herrmann, 2023) adopted the same general structure and a theoretical analysis or broad benchmark of different encoder-decoder combinations has not been done to our knowledge. Neither are we aware of any work that performed a systematic architecture search with the pathway layers or additional fully connected layers on feed-forward VNNs.

#### 5.5.4 Shallow and deep hierarchies

Biologically-informed sparse neural network architectures involve a kind of inductive bias, wherein the hidden layers represent biological entities or processes and the connections between the layers represent biological relations. In principle, such a network can be constructed by starting with a fully-connected network and then applying a masking matrix corresponding to the existence or strength of relations between the biological entities. However, given heterogeneous pathway databases and data processing pipelines, the method of converting a hierarchy of biological pathways into a neural network is by no means standardized. For example, a pathway may contain genes and other pathways: should the network include skip connections to account for this (see Figure 4)? How many hidden layers or nodes in each layer should be chosen before certain paths are “pruned” from the model?

Many architectures incorporate a single layer for pathways following the gene layer, but others extend this to multiple levels to represent a hierarchy of pathways. The methods for constructing these hierarchical relationships vary across models, and often it is not explicitly clear how a “top-level” pathway or biological process is defined. For instance, decisions on whether intermediate pathways should be merged or truncated are typically not well-documented. Ma and Zhang (2019) proposed factor graph neural networks (FGNNs), which include a single pathway layer, but may be “unrolled” to a deeper structure to add greater expressive power.

The early pathway-guided neural network architectures are rather complex, incorporating deep pathway hierarchies. ParsVNN (Huang et al., 2021) applied proximal alternative linearized minimization to the NP-hard problem of *l*_0_ norm and group lasso regularization in order to prune redundant edges between pathways, resulting in simpler structures.

Other authors argue for the simplicity and interpretability of a shallow network (Chen H.-I. H. et al., 2018; Voigt et al., 2024) with just one gene layer aggregating inputs from one or more omics levels, followed by a single pathway layer.

One technical consideration when building a deeper network is how to handle pathways that are not all the same depth of hierarchy. The bottom of a pathway ontology is not well defined, so networks such as P-Net (Elmarakeby et al., 2021) and later Cancer-Net (Pedersen et al., 2023) start from the most abstract level of “biological processes,” defined as Reactome pathways with no parents, successively adding layers for each generation of child pathways, stopping at a user-selected depth, for example, 6 layers. If a branch of the Reactome tree does not have so many levels, then “dummy nodes” are added to allow implementation in a layered machine learning framework like PyTorch. However, since each of these dummy nodes has a nonlinear activation, some information is inevitably lost or altered. In contrast, DrugCell (Kuenzi et al., 2020) employs direct skip connections, which may be more challenging to implement in common ML frameworks but offer greater flexibility in modeling complex biological relationships.

The notion of skip connections can be extended further. Whereas a VNN is typically formulated as a directed acyclic graph with discrete sequential layers, Evans et al. (2024) recently proposed “graph-structured neural networks” (GSNNs)—not to be confused with GNNs—an extension of VNNs that allows for cycles and self-loops. Like GNNs, GSNNs use an input graph as an inductive bias to constrain the information flow in the neural network. Unlike GNNs, GSNNs do not share weights across nodes; instead, each node is associated with its own distinct neural network.

### 5.6 Scientific discovery

VNNs being more performant than their densely-connected counterparts raises the possibility of discovering pathway structures through regularization. That is, if a VNN really is better than an equivalently sparse model, then neural architecture search, for example via pruning, could in principle learn such a structure without prior knowledge integration (Sprang et al., 2024). Such an approach to scientific knowledge discovery—which would enable prediction of new pathway relations, akin to knowledge graph edge prediction—has not yet been widely explored in the VNN literature.

Though ParsVNN (Huang et al., 2021) prunes irrelevant pathways, it starts with a biologically-informed structure. Similarly DeepHisCoM (Park et al., 2022) and DeepBinn (Meirer et al., 2024) explore non-linear relationships only among existing pathways. PathExpSurv (Hou et al., 2023) extended pathway knowledge integration by performing “pathway expansion” to include pathways that may not exist in the original database. First a biologically-sparse network is trained, then fine-tuned with dense connections; weights that are not regularized in the second stage may be indicative of undiscovered pathways. Mixed layers (see Table 3), containing both interpretable and non-interpretable nodes, might allow models to harness predictive performance beyond the constraints of the pathway database, but so far this has not been applied to discovery of new pathways.

### 5.7 Resources and tools

Most papers introducing methods provide open-source code for reproducibility (Appendix Table A4), however, the re-usability and maintainability of some of these codebases is not necessarily guaranteed, especially if raw data or data preprocessing code are not provided or the repository hard-coded for a specific dataset or file system. Nonetheless, Pedersen et al. (2023) was able to reproduce the results of P-Net (Elmarakeby et al., 2021), updating the code to newer ML frameworks. van Hilten et al. (2021) released their GenNet framework, which has since been extended with modules for interpretability (van Hilten et al., 2024b).

A recently developed package called binn (Hartman et al., 2023), focused on proteomics, offers the capability to build VNNs with a given input pathway set. Autoencodix (Joas et al., 2024) is a framework for building different autoencoder architectures, which includes one ontology-based architecture as an option.

There remains a gap, however, for a user-friendly, general-purpose package that supports multi-omics data inputs and different pathway databases and allows exploration of different design decisions to ensure the robustness of the yielded predictions and explanations. Especially interesting would be a highly general package capable of modeling non-biological ontologies (such as in chemistry or social sciences) using a sparse VNN framework.

## 6 Discussion

VNNs have seen increasing adoption since their emergence around 2017–18. Their versatility is evidence in applications spanning protein classification, survival analysis, diagnosis, and drug-interaction prediction (Kulmanov et al., 2018; Hao et al., 2018a,b; Ma et al., 2018). Compared to full-connected neural networks and other ML algorithms, VNNs often demonstrate comparable or superior performance. Studies such as Pedersen et al. (2023) highlight that randomized sparse networks of comparable size underperform in comparison, underscoring the value of integrating biological knowledge. This integration enhances neural networks' ability to extract signals from relatively small and tabular datasets. However, many studies fail to benchmark against traditional ML methods, which can outperform neural networks [e.g., SVMs in Kim and Lee (2023), see also Borisov et al. (2022)], do not compare with non-biologically-informed NNs to quantify the value of knowledge integration, or do not compare their implementation with existing VNN frameworks.

Interpretability is a key advantage of VNNs over “black box” dense neural networks. By constraining networks with biological pathways, VNNs reduce the function space, trading universality for an inductive bias that promotes faster convergence and enables pathway-level insights via node activations (Kerg et al., 2022). Despite this potential, further theoretical exploration of these inductive biases is required. Emerging evidence suggests that node activations in VNNs can be mapped to their biological counterparts to yield novel insights verifiable in the laboratory (e.g., Elmarakeby et al., 2021, validated biomarkers *in vitro*) but no studies have conclusively shown that these activations are both data-driven and shaped by the inductive bias. Sparse network randomizations studies remain insufficient to fully elucidate the function spaces of these models. Additionally, the reliance on simplified gene-to-pathway mappings often neglects directional and regulatory relationships, limiting real-world applicability. Flexible architectures with less restrictive activation functions may better capture the multi-layered complexity of cellular biology, bridging trends in explainability and the training of molecular foundation models (Ma et al., 2024; Hao et al., 2024; Cui et al., 2024).

Another limitation lies in the completeness and quality of pathway databases. While these resources are invaluable, they provide an incomplete picture of cellular processes, particularly for non-human or non-model organisms where database curation is often automated and less robust. Only a few studies, such as Park et al. (2022); Hou et al. (2023), have explored the use of VNNs for pathway expansion or the discovery of new biological relationships, leaving this area largely untapped.

Similarly, the impact of different biological databases or knowledge graphs on performance remains underexplored. Variations in database focus and quality could significantly influence VNN results, but most studies do not benchmark their models across multiple databases.

Finally, nearly all VNN architectures rely on early fusion for multi-omics data, which can result in the loss of structural information and diminished performance. For example, Pedersen et al. (2023) found that fusion strategies had a greater impact on performance than randomized sparse connections. Intermediate fusion techniques, as proposed by Hauptmann and Kramer (2023), may offer a more effective approach. However, there remains a gap in developing VNN architectures that incorporate these advanced strategies.

## 7 Conclusion

Visible neural networks (VNNs) represent a promising advancement in multi-omics data integration, providing a unique combination of predictive performance and biological interpretability. This systematic review has highlighted critical trends, including the importance of sparse architectures informed by biological priors, the impact of design choices on model robustness, and the variability in performance across datasets and ontologies.

While the field has grown substantially since 2020, challenges remain in standardizing terminology, benchmarking methodologies and improving reproducibility. While many works use the nomenclature “Binns” or “VNNs,” other works use different conventions or do not offer any specific name. Some authors test models extensively against other neural networks, both sparse and dense, and classical ML methods, others perform fewer or no such comparisons, motivating a comprehensive benchmarking framework.

Future research should prioritize systematic evaluations of VNN architectures, particularly regarding the robustness of pathway-level interpretations and the interplay between sparsity and prediction accuracy. Expanding the use of pathway databases, integrating multi-omics modalities with more flexible fusion strategies and exploring novel applications beyond oncology could further enhance their utility. Additionally, developing general-purpose, user-friendly software frameworks for constructing and evaluating VNNs would foster broader adoption and reproducibility. Discovering novel pathway relations through neural architecture search remains an untapped opportunity.

By addressing these challenges, VNNs have the potential to unlock novel biological insights, bridge gaps in multi-omics research and contribute to advances in personalized medicine.

## Data Availability

The original contributions presented in the study are included in the article/Supplementary material, further inquiries can be directed to the corresponding authors.
