# Supplementary material for: Visible neural networks for multi-omics integration: a critical review
Source: Front Artif Intell. 2025 Jul 17;8:1595291. doi: 10.3389/frai.2025.1595291 (PMC12310660; doi:10.3389/frai.2025.1595291)
Supplement: Supplementary file 1 [file Table_1.pdf]

1 INCLUDED WORKS

Table A.1 Included VNN papers, identifiers and abbreviations

Abbreviation	Reference	DOI
ACSNI	Anene et al. (2021)	doi:10.1016/j.patter.2021.100270
BiGMLVQ	Voigt et al. (2024)	doi:10.5220/0012420700003657
BINN	Hartman et al. (2023)	doi:10.1038/s41467-023-41146-4
BioM2	Zhang et al. (2024)	doi:10.1093/bib/bbae384
BioVNN	Lin and Lichtarge (2021)	doi:10.1093/bioinformatics/btab137
BioXNet	Yang et al. (2024)	doi:10.1101/2024.01.29.576766
Cancer-Net	Pedersen et al. (2023)	doi:10.48550/arXiv.2309.16645
CellTICS	Yin and Chen (2024)	doi:10.1093/bib/bbad449
consDeepSignaling	Zhang et al. (2021a)	doi:10.3389/fbinf.2021.639349
Cox-PASNet	Hao et al. (2019a)	doi:10.1186/s12920-019-0624-2
DCell	Ma et al. (2018)	doi:10.1038/nmeth.4627
Deep GONet	Bourgeais et al. (2021)	doi:10.1186/s12859-021-04370-7
DeepBINN	Meirer et al. (2024)	doi:10.1109/SDS60720.2024.00044
DeepGAMI	Chandrashekar et al. (2023)	doi:10.1186/s13073-023-01248-6
DeepHisCoM	Park et al. (2022)	doi:10.1093/bib/bbac171
DeepKEGG	Lan et al. (2024)	doi:10.1093/bib/bbae185
DeepOmix	Zhao et al. (2021)	doi:10.1016/j.csbj.2021.04.067
DeepSignalingSynergy	Zhang et al. (2021b)	doi:10.1101/2020.04.10.036491
DeepSigSurvNet	Feng et al. (2021)	doi:10.1186/s12859-020-03850-6
DrugCell	Kuenzi et al. (2020)	doi:10.1016/j.ccell.2020.09.014
DrugCell editorial	Greene and Costello (2020)	doi:10.1016/j.ccell.2020.10.014
DrugCell+fusion	Nguyen et al. (2024)	doi:10.1093/bib/bbae227
DrugVNN	Xie et al. (2024)	doi:10.1101/2024.02.07.579280
DTox	Hao et al. (2022)	doi:10.1016/j.patter.2022.100565
expiMap	Lotfollahi et al. (2023)	doi:10.1038/s41556-022-01072-x
FGNN	Ma and Zhang (2019)	doi:10.48550/arXiv.1906.00537
GCS-Net	Hu et al. (2022)	doi:10.1155/2022/2965166
GeneticNN	Eetemadi and Tagkopoulos (2019)	doi:10.1093/bioinformatics/bty945
GenNet	van Hilten et al. (2021)	doi:10.1038/s42003-021-02622-z
GenNet-Interpret	van Hilten et al. (2024b)	doi:10.1101/2024.02.27.582086
GONN	Peng et al. (2019)	doi:10.1186/s12859-019-2769-6
GraphGONet	Bourgeais et al. (2022)	doi:10.1093/bioinformatics/btac147
GSAE	Chen et al. (2018a)	doi:10.1186/s12918-018-0642-2
GSNNs	Evans et al. (2024)	doi:10.1101/2024.02.28.582164
IRnet	Jiang et al. (2024)	doi:10.1016/j.jare.2024.07.036
k-DNN	Ciallella et al. (2021)	doi:10.1021/acs.est.1c02656
KP-Net	Zhang et al. (2023)	doi:10.1097/CJI.0000000000000475
KPNNs	Fortelny and Bock (2020)	doi:10.1186/s13059-020-02100-5
ME+GE	van Hilten et al. (2024c)	doi:10.1038/s41540-024-00405-w
MiNet	Hao et al. (2019c)	doi:10.1007/978-3-030-20242-2_10

MOViDA	Ferraro et al. (2023)	doi:10.1093/bioinformatics/btad432
MPAC	Liu et al. (2024b)	doi:10.1101/2024.06.15.599113
MPVNN	Ghosh Roy et al. (2022)	doi:10.1093/bioinformatics/btac636
Multilevel-GNN	Yan et al. (2024)	doi:10.1093/bib/bbae184
Multitask-VNN	Zhao et al. (2024)	doi:10.1158/2159-8290.CD-23-0641
NeST-VNN	Park et al. (2024)	doi:10.1038/s43018-024-00740-1
OntoVAE	Doncevic and Herrmann (2023b)	doi:10.1093/bioinformatics/btad387
P-Net	Elmarakeby et al. (2021)	doi:10.1038/s41586-021-03922-4
P-Net Robustness	Esser-Skala and Fortelny (2023)	doi:10.1038/s41540-023-00310-8
PAAE	Avelar et al. (2023)	doi:10.48550/arXiv.2306.05813
PAGE-Net	Hao et al. (2019b)	doi:10.1142/9789811215636_0032
ParsVNN	Huang et al. (2021)	doi:10.1093/nargab/lqab097
PASNet	Hao et al. (2018a)	doi:10.1186/s12859-018-2500-z
PathCNN	Oh et al. (2021)	doi:10.1093/bioinformatics/btab285
PathDeep	Park et al. (2021)	doi:10.3390/ijms222111531
pathDNN	Deng et al. (2020)	doi:10.1021/acs.jcim.0c00331
PathExpSurv	Hou et al. (2023)	doi:10.1186/s12859-023-05535-2
PathME	Lemsara et al. (2020)	doi:10.1186/s12859-020-3465-2
PBAC	Deng et al. (2024)	doi:10.1111/jcmm.18298
PiDeeL	Kaynar et al. (2023)	doi:10.1093/bioinformatics/btad684
PINNet	Kim and Lee (2023)	doi:10.3389/fnagi.2023.1126156
pmVAE	Gut et al. (2021)	doi:10.1101/2021.01.28.428664
priorVAE	Liu et al. (2024a)	doi:10.1371/journal.pcbi.1011198
ReGeNNe	Sharma and Xu (2023)	doi:10.1093/bioinformatics/btad679
Review: Crawford	Crawford and Greene (2020)	doi:10.1016/j.copbio.2019.12.021
Review: Gazestani	Gazestani and Lewis (2019)	doi:10.1016/j.coisb.2019.04.001
Review: Lee	Lee and Kim (2022)	doi:10.3345/cep.2021.01438
Review: Novakovsky	Novakovsky et al. (2023)	doi:10.1038/s41576-022-00532-2
Review: Samal	Samal et al. (2022)	doi:10.3389/fbinf.2022.1036963
Review: van Hilten	van Hilten et al. (2024a)	doi:10.1093/bib/bbae449
Review: Wysocka	Wysocka et al. (2023)	doi:10.1186/s12859-023-05262-8
SigPrimedNet	Gundogdu et al. (2022)	doi:10.1186/s13040-021-00285-4
Varmole	Nguyen et al. (2021)	doi:10.1093/bioinformatics/btaa866
VEGA	Seninge et al. (2021)	doi:10.1038/s41467-021-26017-0
VNNSurv	Tan et al. (2024)	doi:10.1016/j.csbj.2024.07.019
White-Box	Yang et al. (2019b)	doi:10.1016/j.cell.2019.04.016
XAI-AGE	Prosz et al. (2024)	doi:10.1038/s41598-023-50495-5
XMR	Wang et al. (2023)	doi:10.3389/fbinf.2023.1164482

2 DATA AND APPLICATIONS

Table A.2. Disease areas and applications covered in included papers

Disease	n
Cancer	46
Drug Response	13
Cellular Processes	4
Schizophrenia	3
Covid-19	3
Other	15
NA	8

Table A.3. Output formats of neural networks in included papers

Output format	n
Classification	38
Regression	15
Survival	12
Autoencoder	10
Review	7
Survival as Classification	3
Clustering	1
NA	1

3 PRISMA CHECKLISTS

The PRISMA checklist is available to download (`.docx` format): `https://osf.io/pdbk8`

4 CODE AVAILABILITY

Table A.4. Code availability in papers proposing or applying methods

Code available	n	Papers
Yes	62	ACSNI, BINN, BioM2, BioVNN, BioXNet, Cancer-Net, CellTICS, consDeepSignaling, Cox-PASNet, DCell, Deep GONet, DeepGAMI, DeepHisCoM, DeepKEGG, DeepOmix, DeepSigSurvNet, DrugCell, DrugCell+fusion, DrugVNN, DTox, expiMap, GCS-Net, GeneticNN, GenNet, GenNet-Interpret, GraphGONet, GSNNs, IRnet, KP-Net, KPNNs, ME+GE, MiNet, MOViDA, MPAC, MPVNN, Multilevel-GNN, Multitask-VNN, NeST-VNN, OntoVAE, PAAE, PAGE-Net, ParsVNN, PASNet, PathCNN, PathDeep, pathDNN, PathExpSurv, PathME, PBAC, PiDeeL, PINNet, pmVAE, P-Net, P-Net Robustness, priorVAE, ReGeNNe, SigPrimedNet, VEGA, VNNSurv, XAI-AGE, XMR, Varmole
No	7	BiGMLVQ, DeepBINN, DeepSignalingSynergy, FGNN, GONN, GSAE, k-DNN
